# Supplementary material for: Revealing nonlinear neural decoding by analyzing choices
Source: Nat Commun. 2021 Nov 16;12:6557. doi: 10.1038/s41467-021-26793-9 (PMC8595442; doi:10.1038/s41467-021-26793-9)
Supplement: Supplementary file 1 — Supplementary Information [file 41467_2021_26793_MOESM1_ESM.pdf]

Supplementary Information

S.0 Overview

This supplemental material contains mathematical details and proofs of the central ideas presented in the main text.

S.1 Encoding models

- **S.1.1** Orientation estimation task with phase as nuisance
- **S.1.2** Exponential family distributions
- **S.1.3** Quadratic codes
- **S.1.4** Cubic codes

S.2 Information-limiting correlations
S.3 Analyzing decoding quality
S.4 Choice correlations from internal and external sources
S.5 Coarse discrimination and choice correlations
S.6 Orientation variance discrimination task

S.1 Encoding models

S.1.1 Orientation estimation task with varying spatial phase

In Figure 2B, the subject's task is to estimate orientation s near a reference s_0, based on images G of Gabor patterns given by

$$G(\mathbf{x}|s,\nu) = e^{-\|\mathbf{x}\|^2} \cos\left(\mathbf{k}\cdot\mathbf{x}+\nu\right) \tag{1}$$

where $\mathbf{k} = \kappa(\cos s, \sin s)$. Here the target s is the orientation of the pattern, ν is a nuisance variable reflecting the spatial phase, $\mathbf{x}$ is the pixel location in the image, and $\mathbf{k}$ is a spatial frequency vector with amplitude $\kappa = \|\mathbf{k}\|$. We assume the spatial receptive field of simple cell j in primary visual cortex is also described by a Gabor function

$$\mathrm{RF}_j(\mathbf{x}, s_j, \nu_j) = e^{-\|\mathbf{x}\|^2} \cos\left(\mathbf{k}_j\cdot\mathbf{x}+\nu_j\right) \tag{2}$$

$$\mathbf{k}_j = \kappa(\cos s_j, \sin s_j) \tag{3}$$

where each neuron has a preferred orientation s_j, spatial phase ν_j, and spatial frequency $\mathbf{k}_j$. Here for simplicity we assume that all neurons' preferred spatial frequencies have the same amplitude κ that matches the input image.

We model the mean neuronal responses by the overlap between the image and their linear receptive field. This overlap determines the tuning curve of each neuron:

$$\begin{aligned} f_j(s,\nu) &= \int d\mathbf{x}\, G(\mathbf{x}|s,\nu)\mathrm{RF}_j(\mathbf{x},s_j,\nu_j) \\ &= \Big[e^{-\frac{1}{4}\kappa^2\cos(s-s_j)}\cos\left(\nu+\nu_j\right) \\ &\quad + e^{+\frac{1}{4}\kappa^2\cos(s-s_j)}\cos\left(\nu-\nu_j\right)\Big]\,\frac{\pi}{4}e^{-\frac{1}{4}\kappa^2} \end{aligned} \tag{4}$$

This expression can be written in the form:

$$f_j(s,\nu) = A_j(s)\cos\left(\nu+\psi_j(s)\right) \tag{5}$$

using the stimulus-dependent response amplitude

$$A_j(s) = C\sqrt{2\cosh 2\beta_j(s) + 2\cos 2\nu_j} \tag{6}$$

and phase

$$\psi_j(s) = \nu_j - \alpha_j(s) \tag{7}$$

where we define the quantities

$$C = \frac{\pi}{4}\exp\left(-\frac{1}{4}\kappa^2\right) \tag{8}$$

$$\beta_j(s) = \frac{1}{4}\kappa^2\cos(s-s_j) \tag{9}$$

$$\alpha_j(s) = \tan^{-1}\frac{\exp\left(\beta_j(s)\right)\sin 2\nu_j}{\exp\left(-\beta_j(s)\right)+\exp\left(\beta_j(s)\right)\cos 2\nu_j} \tag{10}$$

Equation 5 reveals that the mean response of each neuron traces out a sinusoidal oscillation in ν, where the amplitude and phase depend on s and the specific neuron j. The mean tuning for each pair of neurons therefore traces out an ellipse as a function of the nuisance variable, the input's spatial phase. When we *average* over the ellipse generated by the nuisance

variable ν, the mean tuning to s is abolished — but the response *covariances* (nuisance correlations) remain tuned to s.

Assuming each neuron's response variability is drawn independently from a standard Gaussian $\mathcal{N}(0,1)$, we can write the response distribution as

$$P(\mathbf{r}|\nu, s) = \mathcal{N}(\mathbf{f}(s,\nu), \mathbf{I}) \tag{11}$$

If the spatial phase ν were fixed and known, the brain could estimate the orientation just from the mean tuning of the neural responses. However, if the spatial phase is unknown and varies between stimulus presentations uniformly from 0 to 2π, the mean tuning $\mathbf{f}(s)$ can be expressed as

$$f_j(s) = \langle r_j|s\rangle = \int r_j\, p(r_j|s) dr_j \tag{12}$$
$$= \iint r_j\, p(r_j|s,\nu)\, p(\nu)\, dr_j\, d\nu \tag{13}$$
$$= \int f_j(s,\nu) p(\nu)\, d\nu \tag{14}$$
$$= \frac{1}{2\pi}\int f_j(s,\nu)\, d\nu \tag{15}$$
$$= \frac{A_j(s)}{2\pi}\int_0^{2\pi} \cos\left(\nu + \psi_j(s)\right) d\nu \tag{16}$$
$$= 0 \tag{17}$$

This shows that there is no signal in the mean responses.

However, the brain can perform quadratic computations to eliminate the nuisance variable. We can define $\mathrm{Cov}_{ij}[\mathbf{r}|s,\nu]$ as the neural covariance (noise correlations) when everything in the image is fixed, and $\mathrm{Cov}_{ij}[\mathbf{r}|s]$ as the neural covariance when the nuisance is unknown and free to vary (nuisance correlations). Then $\mathrm{Cov}_{ij}[\mathbf{r}|s]$ is

$$\mathrm{Cov}_{ij}[\mathbf{r}|s] = \langle (r_i - f_i(s))(r_j - f_j(s))|s\rangle \tag{18}$$
$$= \langle r_i r_j|s\rangle = \iint r_i r_j\, p(\mathbf{r}|s) dr_i dr_j \tag{19}$$
$$= \int d\nu \iint r_i r_j\, p(\mathbf{r}|s,\nu) p(\nu) dr_i dr_j \tag{20}$$
$$= \int d\nu\, p(\nu) \langle r_i r_j|s,\nu\rangle \tag{21}$$
$$= \int d\nu\, p(\nu)\left(\mathrm{Cov}_{ij}[\mathbf{r}|s,\nu] + f_i(s,\nu) f_j(s,\nu)\right) \tag{22}$$
$$= \frac{1}{2\pi}\delta_{ij} + \frac{1}{2\pi}\int d\nu f_i(s,\nu) f_j(s,\nu) \tag{23}$$
$$= \frac{1}{2\pi}\delta_{ij} + \frac{1}{2\pi} D_{ij}(s) \tag{24}$$

where $D_{ij}(s)$ is given by

$$\begin{aligned} D_{ij}(s) &= \int d\nu f_i(s,\nu) f_j(s,\nu) \\ &= \int d\nu\, A_i(s)\cos\left(\nu + \psi_i(s)\right) A_j(s) \cos\left(\nu + \psi_j(s)\right) \\ &= \pi \cos(\psi_i(s) - \psi_j(s)) A_i(s) A_j(s) \end{aligned} \tag{25}$$

Here when we compute Equation 25, we used the trigonometric identity: $2\cos(x)\cos(y) = \cos(x+y) + \cos(x-y)$, and $\int \cos(2\nu + \psi_i + \psi_j) d\nu = 0$.

This demonstrates that the neural covariance $\mathrm{Cov}_{ij}[\mathbf{r}|s]$ depends on the orientation s. While linear computation is useless for estimating orientation since the mean responses are untuned (12), quadratic (or higher-order) nonlinear computations can be used to estimate the orientation.

S.1.2 Exponential family distributions

For a stimulus s and a response $\mathbf{r}$, the conditional probability is a member of the exponential family when

$$p(\mathbf{r}|s) = b(\mathbf{r}) \exp\Big(\mathbf{\Theta}(s)^\top \mathbf{R}(\mathbf{r}) - A(s)\Big) \tag{26}$$

where $\mathbf{\Theta}(s)$ are the natural parameters, $\mathbf{R}(\mathbf{r})$ are the sufficient statistics, $A(s)$ and $b(\mathbf{r})$ are the log normalizer and base measure. The statistics $\mathbf{R}(\mathbf{r})$ are called sufficient because they contain all the information needed to estimate the stimulus s.

S.1.2.1 Fisher information

One measure of information content that a population response contains about a stimulus is the Fisher information $J(s)$ [1–3,6–8]. The Fisher information is given by

$$J = -\left\langle \frac{\partial^2}{\partial s^2} \log p(\mathbf{r}|s)\right\rangle_{\mathbf{r}|s} \tag{27}$$
$$= \left\langle \left(\frac{\partial}{\partial s}\log p(\mathbf{r}|s)\right)^2\right\rangle_{\mathbf{r}|s} \tag{28}$$

For distributions $p(\mathbf{r}|s)$ in the exponential family with sufficient statistics $\mathbf{R}(\mathbf{r})$, we can compute these quantities analytically. We denote the mean of the sufficient statistics as $\mathbf{F}(s) = \langle \mathbf{R}(\mathbf{r})|s\rangle$. This mean $\langle \mathbf{R}|s\rangle$ can be obtained by differentiating $A(s)$ by the natural parameters $\mathbf{\Theta}(s)$,

$$\mathbf{F} = \frac{\partial A(s)}{\partial \mathbf{\Theta}(s)} \tag{29}$$

Equation 29 can give us the first and second derivatives of $A(s)$ over s.

$$A' = \sum_i \frac{\partial A}{\partial \Theta_i} \frac{d\Theta_i}{ds} = \boldsymbol{\Theta}'^\top \mathbf{F} \tag{30}$$

$$A'' = \boldsymbol{\Theta}''^\top \mathbf{F} + \boldsymbol{\Theta}'^\top \mathbf{F}' \tag{31}$$

Thus we can compute two definitions of Fisher information.

$$J = -\left\langle \frac{\partial^2}{\partial s^2} \log P(\mathbf{r}|s) \right\rangle_{P(\mathbf{r}|s)} \tag{32}$$

$$= A'' - \boldsymbol{\Theta}''^\top \mathbf{F} \tag{33}$$

$$= \boldsymbol{\Theta}'^\top \mathbf{F}' \tag{34}$$

and

$$J = \left\langle \left(\frac{\partial}{\partial s} \log P(\mathbf{r}|s) \right)^2 \right\rangle_{P(\mathbf{r}|s)} \tag{35}$$

$$= \boldsymbol{\Theta}'^\top (\langle \mathbf{R}\mathbf{R}^\top \rangle - \mathbf{F}\mathbf{F}^\top) \boldsymbol{\Theta}' \tag{36}$$

$$= \boldsymbol{\Theta}'^\top \Gamma \boldsymbol{\Theta}' \tag{37}$$

where $\Gamma = \mathrm{Cov}[\mathbf{R}(\mathbf{r})|s]$.

Since the two definition are equivalent, we have

$$\boldsymbol{\Theta}' = \Gamma^{-1} \mathbf{F}' \tag{38}$$

Substituting Equation 38 into Equation 37, we find the Fisher Information for the exponential family [5]

$$J = \mathbf{F}'^\top \Gamma^{-1} \mathbf{F}' \tag{39}$$

S.1.2.2 Optimal estimation in the exponential family

Again assuming responses come from this distribution, we want to compute the maximum likelihood stimulus, $\hat{s}$, near a reference stimulus s_0:

$$\hat{s} = \underset{s}{\mathrm{argmax}}\; p(\mathbf{r}|s) \tag{40}$$

$$= \underset{s}{\mathrm{argmax}}\; \log p(\mathbf{r}|s) \tag{41}$$

$$= \underset{s}{\mathrm{argmax}}\; \boldsymbol{\Theta}(s)^\top \mathbf{R}(\mathbf{r}) - A(s) \tag{42}$$

A Taylor expansion around the reference yields

$$\begin{aligned} &\boldsymbol{\Theta}(s)^\top \mathbf{R}(\mathbf{r}) - A(s) \\ &\quad \approx [\boldsymbol{\Theta}^\top \mathbf{R} - A] \\ &\quad + [\boldsymbol{\Theta}'^\top \mathbf{R} - A'](s - s_0) \\ &\quad + \tfrac{1}{2}(s - s_0)^\top [\boldsymbol{\Theta}''^\top \mathbf{R} - A''](s - s_0) + \cdots \end{aligned} \tag{43}$$

where all functions and derivatives are evaluated at s_0. We find the maximum $\hat{s}$ by differentiating with respect to s and setting the result equal to zero:

$$0 = [\boldsymbol{\Theta}'^\top \mathbf{R} - A'] + (\hat{s} - s_0)[\boldsymbol{\Theta}''^\top \mathbf{R} - A''] \tag{44}$$

The solution is

$$\hat{s} = s_0 - \frac{\boldsymbol{\Theta}'^\top \mathbf{R} - A'}{\boldsymbol{\Theta}''^\top \mathbf{R} - A''} \tag{45}$$

Since $\mathbf{r}$ is a random quantity, we can express $\mathbf{R}$ as a mean and a deviation away from that mean: $\mathbf{R} = \langle \mathbf{R}|s_0 \rangle + \delta\mathbf{R} = \mathbf{F} + \delta\mathbf{R}$. In this case, $\boldsymbol{\Theta}''^\top \mathbf{R} - A'' = \boldsymbol{\Theta}''^\top \mathbf{F} - A'' + \boldsymbol{\Theta}''^\top \delta\mathbf{R}$, where the mean term is precisely the negative Fisher Information $-J(s_0)$. If the trial-to-trial fluctuations in the uncertainty are small relative to the average uncertainty then this Fisher term will dominate. Then we have

$$\hat{s} = \mathbf{w}^\top \mathbf{R} + \mathbf{c} \tag{46}$$

where

$$\mathbf{w} = \frac{\boldsymbol{\Theta}'}{J} = \frac{\Gamma^{-1}\mathbf{F}'}{\mathbf{F}'^\top \Gamma^{-1} \mathbf{F}'} \tag{47}$$

and where we used the results from Equations 39 and 38, with $\Gamma = \mathrm{Cov}(\mathbf{R}|s_0)$ and $\mathbf{F} = \langle \mathbf{R}|s_0 \rangle$. Thus, in this limit, the optimal estimator for s is a linear decoding of the sufficient statistics $\mathbf{R}(\mathbf{r})$.

S.1.3 Quadratic codes

In a purely quadratic coding model (no linear information), the distribution of neural responses is described by the exponential family with quadratic sufficient statistics, $p(\mathbf{r}|s) \sim \exp[\boldsymbol{\Theta}(s)^\top \mathbf{R}(\mathbf{r})]$ where $\mathbf{R}(\mathbf{r}) = (\ldots, r_i r_j, \ldots)$. A familiar example is a Gaussian distribution with stimulus-dependent covariance: $p(\mathbf{r}|s) = N(\mathbf{f}, \Sigma(s))$.

As a concrete example we construct a covariance that rotates with stimulus s. Any covariance matrix needs to be positive semidefinite. We build $\Sigma(s)$ by setting the eigenvalues to be positive and s-independent and eigenvectors to form an orthogonal basis that rotates with s:

$$\Sigma(s) = V(s) \Lambda V(s)^\top \tag{48}$$

where $V(s) = \exp As$ is a rotation matrix in which $A = -A^\top$ is a real antisymmetric matrix with pure imaginary eigenvalues, and Λ is a diagonal matrix composed of all positive eigenvalues of $\Sigma(s)$.

To calculate the Fisher Information (Equation 39), we need to first calculate the derivative of the mean

$\mathbf{F}' = \frac{\partial}{\partial s} \langle \mathbf{R}(\mathbf{r}) | s \rangle$ and covariance $\Gamma = \mathrm{Cov}[\mathbf{R}(\mathbf{r})|s]$ of the quadratic sufficient statistics.

Because the mean of $\mathbf{r}$ is not dependent on the stimulus in this example, we can compute $F'_{ij} = \langle r_i r_j | s \rangle' = \Sigma'_{ij}(s)$, where $\Sigma'_{ij}(s)$ is the derivative of the covariance of $\mathbf{r}$,

$$\Sigma'(s) = U e^{\Omega s} (\Omega X - X \Omega) e^{-\Omega s} U^\dagger \tag{49}$$

where $\dagger$ denotes a conjugate transpose. Here Ω is a diagonal matrix of eigenvalues for A, U is an orthogonal matrix of the eigenvectors of A, and $X = U^\dagger \Lambda U$.

The elements in Γ can be expressed as $\Gamma_{ij,kn} = \langle r_i r_j r_k r_n | s \rangle - \langle r_i r_j | s \rangle \langle r_k r_n | s \rangle$. We can use the following identity for a Gaussian to compute this fourth-order quantity:

$$\begin{aligned} \langle r_i r_j r_k r_n | s \rangle = & \langle r_i r_j | s \rangle \langle r_k r_n | s \rangle + \langle r_j r_n | s \rangle \langle r_i r_k | s \rangle \\ & + \langle r_i r_n | s \rangle \langle r_j r_k | s \rangle \end{aligned} \tag{50}$$

where

$$\langle r_i r_j | s \rangle = \Sigma_{ij} + f_i f_j \tag{51}$$

Substitution of the response covariance (Equation 48) into Equation 50 allows us to calculate the covariance Γ of the quadratic sufficient statistics, and thereby to estimate the stimulus and Fisher information for this quadratic code.

S.1.4 Cubic codes

In Figure S1 we assume the brain encodes the stimulus using a cubic code. A simple cubic code in $\mathbf{z} = (z_i, z_j, z_k) \in \mathbb{R}^3$ can be written as

$$p(\mathbf{z}|s) = \tfrac{1}{Z} \exp\left(\gamma(s) z_i z_j z_k - \|\mathbf{z}\|^4 \right) \tag{52}$$

where we include the base measure $e^{-\|\mathbf{z}\|^4}$ to ensure normalizability (Figure S1A).

For mathematical convenience, we approximate this code by a mixture of Gaussians.

$$\begin{aligned} p(\mathbf{z}|s) &\approx \sum_{a=1}^{4} p(a) p(\mathbf{z}|a, s) && (53) \\ &= \sum_a \frac{1}{4} \mathcal{N}(\mathbf{z}|\mu_a(s), M_a(s)) && (54) \end{aligned}$$

where

$$\mathbf{m}_a(s) = \frac{s}{\sqrt{1+s^2}} \mathbf{v}_a \tag{55}$$

and

$$M_a(s) = \frac{I + s^2 \mathbf{v}_a \mathbf{v}_a^\top}{(1+s^2)^2} \tag{56}$$

The vectors $\mathbf{v}_a$ reflect the four corners of the tetrahedron, $v_{a,i} = \pm 1$, to match the tetrahedral symmetry of the pure cubic code (Equation 52, Figure S1). To sample from this distribution, we randomly choose a component a and then sample from the gaussian $\mathcal{N}(\mathbf{z}|\mathbf{m}_a(s), M_a(s))$ conditioned on that component.

This distribution has zero mean and identity covariance but a nontrivial skewness tensor, and qualitatively matches the corresponding distribution for the true exponential family distribution with cubic sufficient statistics (Figure S1).

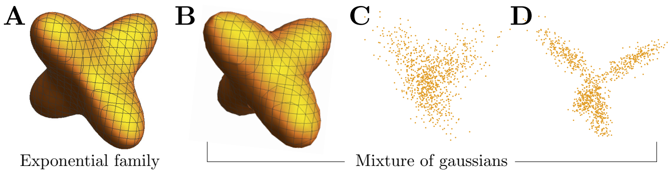

Figure S1: Multivariate skewed distributions. (**A**) Isoprobability contour of an exponential family distribution with cubic statistics in three dimensions, drawn from $p(\mathbf{z}|s) \propto \exp\left(s z_1 z_2 z_3 - \|\mathbf{z}\|^4\right)$. (**B**) Isoprobability contour for a mixture of four gaussians (Eq. 54). (**C,D**) Samples drawn from the mixture form, with $s = 1, 2$.

For simplicity, we consider pure cubic codes with non-overlapping cliques of three variables.

$$p(\mathbf{z}|s) = \prod_\alpha p(\mathbf{z}_\alpha|s) = \prod_\alpha p(z_{\alpha_1}, z_{\alpha_2}, z_{\alpha_3}|s) \tag{57}$$

To convert this purely cubic distribution into a distribution with linear and quadratic information as well, we simply shift and scale the distribution in a manner dependent on s:

$$\begin{aligned} \mathbf{r} &= \mathbf{f}(s) + \Sigma^{1/2}(s)\, \mathbf{z}\, \Sigma^{1/2}(s) && (58) \\ \mathbf{z} &\sim \frac{1}{Z(s)} \exp\left[\sum_{ijk} \gamma_{ijk}(s) z_i z_j z_k - \|\mathbf{z}\|^4 \right] && (59) \end{aligned}$$

These affine transformations can be incorporated directly into each component of the mixture of gaussians,

$$p(\mathbf{r}|a, s) = \mathcal{N}(\mathbf{r}|\mathbf{f}(s) + \mathbf{m}_a(s), \Sigma^{1/2}(s) M_a(s) \Sigma^{1/2}(s)) \tag{60}$$

Note that the linear and quadratic information terms are independent of the component a.

S.2 Information-limiting correlations

Information-limiting correlations [3] describe variability that cannot be averaged away because they are indistinguishable from changes in the stimulus. These fluctuations can ultimately be referred back to the stimulus, to appear as $\mathbf{r} \sim p(\mathbf{r}|s + ds)$, where ds is zero mean noise with variance $1/J_\infty$ which determines the uncertainty of stimulus. Applying the law of total covariance, we can decompose the covariance of nonlinear statistics $\mathbf{R}(\mathbf{r})$ conditioned on the stimulus into two parts:

$$\begin{aligned}\Gamma =& \mathrm{Cov}(\mathbf{R}(\mathbf{r})|s) \\ =& \langle \mathrm{Cov}(\mathbf{R}(\mathbf{r})|s, ds)\rangle_{ds} + \mathrm{Cov}\,\langle \mathbf{R}(\mathbf{r})|s, ds\rangle_{\mathbf{r}}\end{aligned} \tag{61}$$

where $\langle\cdot\rangle$ indicates an expectation value over the subscripted variable. The first term can be computed as follows,

$$\langle \mathrm{Cov}(\mathbf{R}(\mathbf{r})|s, ds)\rangle_{ds} = \langle \Gamma(s + ds)\rangle_{ds} \tag{62}$$
$$\approx \langle \Gamma_0 + ds\,\Gamma'\rangle_{ds} \tag{63}$$
$$= \Gamma_0 \tag{64}$$

Here we denote the covariance of $\mathbf{R}(\mathbf{r})$ given s and ds as $\Gamma(s+ds)$. The second equality used a Taylor expansion of $\Gamma(s+ds)$ around s. The third equality used the fact that the mean of ds is zero. Γ_0 is the covariance of $\mathbf{R}$ in the absence of information-limiting correlations. The second term in Equation 61 can be expressed as

$$\mathrm{Cov}\,\langle \mathbf{R}(\mathbf{r})|s, ds\rangle_{\mathbf{r}} \tag{65}$$
$$= \mathrm{Cov}(\mathbf{F}(s + ds)|s) \tag{66}$$
$$\approx \mathrm{Cov}(\mathbf{F}(s) + ds\,\mathbf{F}'(s)|s) \tag{67}$$
$$= \frac{1}{J_\infty}\mathbf{F}'(s)\mathbf{F}'(s)^\top \tag{68}$$

Here we have written the mean of $\mathbf{R}(\mathbf{r})$ given s and ds as $\mathbf{F}(s + ds)$. The second equality used a first-order expansion of $\mathbf{F}(s + ds)$ around s. The third equality used the fact that the variance of ds is $1/J_\infty$.

Equation 61 can therefore be written as

$$\Gamma = \Gamma_0 + \frac{1}{J_\infty}\mathbf{F}(s)'\mathbf{F}(s)'^\top \tag{69}$$

which is a rank-one perturbation of the covariance Γ_0.

To compute the nonlinear Fisher Information, $J_{R(\mathbf{r})} = \mathbf{F}'^\top\Gamma^{-1}\mathbf{F}'$, we can use the Sherman-Morrison lemma to compute Γ^{-1}:

$$\Gamma^{-1} = \Gamma_0^{-1} - \frac{\Gamma_0^{-1}\mathbf{F}'\mathbf{F}'^\top\Gamma_0^{-1}}{J_\infty + \mathbf{F}'\Gamma_0^{-1}\mathbf{F}'^\top} \tag{70}$$

Substituting these equations into the nonlinear Fisher Information (Equation 39) and simplifying, we obtain

$$J_{R(\mathbf{r})} = \frac{1}{1/J_\infty + 1/J_0} \tag{71}$$

Here $J_0 = \mathbf{F}'^\top\Gamma_0^{-1}\mathbf{F}'$ is the nonlinear Fisher Information in the absence of information-limiting correlations. When the population size grows, the term J_0 grows proportionally [1, 2], so for large populations the output information saturates at J_∞.

S.3 Analyzing decoding quality

S.3.1 Unknown nonlinearities

The true nonlinearity that the brain uses to estimate the stimulus is unknown. Thus a crucial question in our decoding analysis is, which nonlinearities to consider? One reasonable set is polynomials in $\mathbf{r}$, *i.e.* a Taylor series expansion of the neural nonlinearities, $\mathbf{\Psi}(\mathbf{r}) = (r_i, r_ir_j, r_ir_jr_k, ...)$.

The locally optimal decoder is a weighted sum of the sufficient statistics $\mathbf{R}(\mathbf{r})$ (Equation 46):

$$\hat{s}_{\mathrm{opt}} = \mathbf{w}\cdot\mathbf{R}(\mathbf{r}). \tag{72}$$

However, the brain might choose a different nonlinear basis $\mathbf{g}(\mathbf{r})$:

$$\hat{s}_{\mathrm{brain}} = \mathbf{v}\cdot\mathbf{g}(\mathbf{r}). \tag{73}$$

As long as the brain's nonlinear function spans the same function basis as the sufficient statistics, we can still get all of the information about stimulus from neural population. This allows us to use choice correlation between brain's estimate $\hat{s}_{\mathrm{brain}}$ and our analysis nonlinearity $\Psi(\mathbf{r})$ to check the optimality condition (Equation 7).

In Figure 4, we assumed that the optimal nonlinear basis function $\mathbf{R}$ is polynomial nonlinearity up to third order, $\mathbf{R}(\mathbf{r}) = (r_i, r_ir_j, r_ir_jr_k, ...)$. We used cubic codes described in Methods **Cubic encoding** to generate neural responses for which $\mathbf{R}(\mathbf{r})$ are sufficient statistics for the stimulus. In this simulation, 18 neuronal responses (six cliques of size 3) were generated using cubic codes.

Our model brain decodes the stimulus using a cascade of linear-nonlinear transformations, with Rectified Linear Units ($\mathrm{ReLU}(x) = \max(0, x)$) for the nonlinear activation functions. We used a fully-connected ReLU network with two hidden layers and 30 units per hidden layer,

$$\hat{s}_{\text{brain}} = \mathbf{v} \cdot \mathbf{r}^{(3)} + \mathbf{b}^{(3)} \tag{74}$$

$$\mathbf{r}^{(3)} = \mathrm{ReLU}(\mathbf{W}^{(2)}\mathbf{r}^{(2)} + \mathbf{b}^{(2)}) \tag{75}$$

$$\mathbf{r}^{(2)} = \mathrm{ReLU}(\mathbf{W}^{(1)}\mathbf{r}^{(1)} + \mathbf{b}^{(1)}) \tag{76}$$

$$\mathbf{r}^{(1)} = \mathbf{r} \tag{77}$$

We trained the neural network with 20000 response samples generated from a cubic code driven by stimuli near the reference s_0. We optimized the estimation performance for the neural network using backpropagation to find weights $\{\mathbf{W}^{(\ell)}\}$, biases $\{\mathbf{b}^{(\ell)}\}$, and readout vector $\mathbf{v}$ that minimized the mean squared error. Our trained neural network performed near-optimally, extracting 91% of the Fisher information compared to optimal decoding based on the true sufficient statistics.

Feigning ignorance of our simulated brain's true decoder, we applied the nonlinear choice correlation test (Equation 7) using monomial nonlinearities $\Psi(\mathbf{r})$ up to third order, *e.g.* r_i, $r_i r_j$, $r_i^2 r_j$, r_k^3, etc. The simulated choice correlations were calculated by Equation 5, where $\mathbf{R}(\mathbf{r}) = \Psi(\mathbf{r})$ based on neural responses driven by the reference stimulus s_0, and the stimulus estimate was $\hat{s}_{\text{brain}}$. The optimal choice correlation is computed using Equation 7, where $\sqrt{J_{\Psi(\mathbf{r})}} = d'_{\Psi}/\Delta s = \frac{\Delta \mathbf{F}_{\Psi}}{\Delta s\, \sigma_{\Psi}}$, and $\sqrt{J} \approx 1/\sigma_{\hat{s}_{\text{brain}}}$. We computed $\Delta \mathbf{F}_{\Psi}$ based on neural population responses $\mathbf{r}_+$ and $\mathbf{r}_-$ driven by stimuli $s_+ = s_0 \pm \Delta s/2$. The change in mean was $\Delta \mathbf{F}_{\Psi} = \langle \Psi(\mathbf{r}_+) \rangle - \langle \Psi(\mathbf{r}_-) \rangle$, and the average variance was $\sigma_{\Psi}^2 = \frac{1}{2}\mathrm{Var}(\Psi(\mathbf{r}_+)) + \frac{1}{2}\mathrm{Var}(\Psi(\mathbf{r}_-))$. The trained neural network's estimate $\hat{s}_{\text{brain}}$ has a variance $\sigma^2_{\hat{s}_{\text{brain}}}$ near the reference stimulus s_0. Based on these quantities, Figure 4 shows that we can successfully identify that the brain is near-optimal.

S.3.2 Decoding efficiency

A decoder that would be suboptimal for one population code could be near-optimal in the presence of information-limiting noise. In this case, nonlinear choice correlations can be decomposed into a sum of two terms, one from the information-limiting component and the other from the rest of the noise [9]:

$$C_{R_k} = \frac{(\Gamma \mathbf{w})_k}{\sigma_k \sigma_{\hat{s}}} = \frac{(\Gamma_0 \mathbf{w} + \frac{1}{J_\infty}\mathbf{F}'\mathbf{F}'^{\top}\mathbf{w})_k}{\sigma_k \sigma_{\hat{s}}} \tag{78}$$

For unbiased decoding, $\mathbf{w}^{\top}\mathbf{F}' = 1$. Some manipulation gives [9]

$$C_{R_k} = \frac{(\Gamma_0 \mathbf{w})_k}{\Gamma_{0k}\sigma_{0\hat{s}}} \frac{\sigma_{0\hat{s}}}{\sigma_{\hat{s}}} \frac{\Gamma_{0k}}{\Gamma_k} + \frac{F'_k}{\sigma_k}\sigma_{\hat{s}} \frac{1/J_\infty}{\sigma_{\hat{s}}^2} \tag{79}$$

where $\Gamma_{0k} = (\Gamma_0)_{kk} \approx \Gamma_{kk}$ for small information-limiting noise variance $1/J_\infty \ll \Gamma_{0k}$ (which nonetheless can have a large effect on information despite the small variance), and where $\sigma_{0\hat{s}}$ is the standard deviation of the estimate produced by the same suboptimal decoder $\mathbf{w}$ in the absence of information-limiting correlations, *i.e.* when the covariance of the sufficient statistics is Γ_0. The variance of $\hat{s}$ can itself be decomposed into two terms as well:

$$\sigma_{\hat{s}}^2 = \mathbf{w}^{\top}\Gamma\mathbf{w} = \mathbf{w}^{\top}\Gamma\mathbf{w} + \frac{1}{J_\infty}\mathbf{w}^{\top}\mathbf{F}'\mathbf{F}'^{\top}\mathbf{w} \tag{80}$$

$$= \sigma_{0\hat{s}}^2 + 1/J_\infty \tag{81}$$

where we assume unbiased decoding, which implies $\mathbf{w}^{\top}\mathbf{F}' = 1$. This expression allows us to represent the ratio $\frac{\sigma_{0\hat{s}}}{\sigma_{\hat{s}}}$ as

$$\frac{\sigma_{0\hat{s}}}{\sigma_{\hat{s}}} = \sqrt{1 - \frac{1/J_\infty}{\sigma_{\hat{s}}^2}} = \sqrt{1-\alpha} \tag{82}$$

with $\alpha = \frac{1/J_\infty}{\sigma_{\hat{s}}^2}$. Substituting these into (Eq. 79) we find that the choice correlation for a suboptimal decoder in the presence of information-limiting correlations is a weighted sum of the choice correlations for optimal and suboptimal decoding:

$$C_R^{\text{sub}} \approx \alpha C_R^{\text{opt}} + C_R^{\text{sub}}\sqrt{1-\alpha} \tag{83}$$

Here C_R^{sub} and C_R^{opt} are, respectively, the choice correlations for suboptimal decoding without information-limiting noise (so $\Gamma = \Gamma_0$), and choice correlations for optimal decoding.

The slope α between choice correlations and those predicted from optimal decoding is equal to the fraction of estimator variance explained by information-limiting noise. This slope therefore provides an estimate of the efficiency of the brain's decoding.

S.4 Choice correlations from internal versus external noise

The response covariance that drives fluctuations in choices could arise from internal or external (nuisance) variability, or both. Choice correlations predicted for optimal decoding differ depending on whether we condition on the nuisance variables or not. In the main text, we described optimal choice correlations under the distribution $p(\mathbf{r}|s)$. This includes variations caused by external nuisance variables, which is sensible since this is what the brain's decoder must handle. However, it is also potentially informative to examine how purely internal variability correlates with choice, as this is often how choice correlations are assessed. In this section, we derive the choice correlations driven by purely internal noise, for a decoder that learned to remove external nuisance variation as well.

For simplicity we assume that the nonlinear sufficient statistics $\mathbf{R}(\mathbf{r})$ are linearly tuned to both the stimulus s and a scalar nuisance variable ν,

$$\mathbf{R}(\mathbf{r}) = \mathbf{F}'s + \mathbf{G}'\nu + \eta \tag{84}$$

where $\mathbf{F}'$ and $\mathbf{G}'$ characterize the sensitivity of $\mathbf{R}(\mathbf{r})$ to stimulus s and nuisance ν, and an internal noise source η has zero mean with covariance H. We assume the brain has a prior over the nuisance variation, $p(\nu)$, with zero mean and variance ξ. The total covariance for internal and external fluctuations is then

$$\Gamma = H + \xi\mathbf{G}'\mathbf{G}'^{\top} \tag{85}$$

When we measure choice correlations while fixing the nuisance variables in the experiment, we assume the brain retains its decoding strategy accounting for both internal noise and unknown nuisance variation, and not the optimal decoding strategy when the nuisance is fixed and known. These decoding weights are

$$\mathbf{w} = \frac{\Gamma^{-1}\mathbf{F}'}{J_1} \tag{86}$$

where the denominator $J_1 = \mathbf{F}'^{\top}\Gamma^{-1}\mathbf{F}'$ is the Fisher information about s when there is natural nuisance variation following $p(\nu)$. For distributions in the exponential family, this information saturates the Cramer-Rao bound on an estimator's variance, so that $J_1 = 1/\sigma_{\hat{s}}^2$ [12]. The normalization by J_1 ensures the decoding is locally unbiased. These weights are used to estimate the stimulus according to

$$\hat{s} = \mathbf{w}^{\top}\mathbf{R}(\mathbf{r}) + b \tag{87}$$

Choice correlations in this fixed-nuisance experiment will be denoted by a lowercase c:

$$c_{R_k}^{\text{sub}} = \text{Corr}(R_k, \hat{s}|s, \nu) \tag{88}$$

We include the superscript c^{sub} as a reminder that these choice correlations do not follow the optimal pattern when the decoder is not matched to only the purely internal variability, as here.

We can express these choice correlations as:

$$c_{R_k}^{\text{sub}} = \frac{\text{Cov}(R_k, \hat{s}|s, \nu)}{\sigma_{R_k|s,n}\sigma_{\hat{s}|s,n}} \tag{89}$$

The covariance between $\hat{s}$ and $\mathbf{R}$ is

$$\begin{aligned} \text{Cov}(\mathbf{R}, \hat{s}|s, \nu) &= \langle \mathbf{R}\hat{s}|s, n\rangle & (90)\\ &= \langle \mathbf{R}\mathbf{R}^{\top}|s, n\rangle\mathbf{w} & (91)\\ &= \frac{H\Gamma^{-1}\mathbf{F}'}{J_1} & (92) \end{aligned}$$

For the scalar nuisance variable we assume here, we can use the Sherman-Morrison lemma to decompose the inverse of the total covariance into a rank-one perturbation of the internal noise inverse covariance:

$$\begin{aligned} \Gamma^{-1} &= (H + \xi\mathbf{G}'\mathbf{G}'^{\top})^{-1} & (93)\\ &= H^{-1} - \frac{H^{-1}\mathbf{G}'\mathbf{G}'^{\top}H^{-1}}{1/\xi + \mathbf{G}'^{\top}H^{-1}\mathbf{G}'} & (94) \end{aligned}$$

Substituting this inverse covariance into Equation 90, we obtain

$$\begin{aligned} &\text{Cov}(\mathbf{R}, \hat{s}|s, \nu) & (95)\\ &= \frac{1}{J_1}H(H^{-1} - \frac{H^{-1}\mathbf{G}'\mathbf{G}'^{\top}H^{-1}}{1/\xi + \mathbf{G}'H^{-1}\mathbf{G}'^{\top}})\mathbf{F}' & (96)\\ &= \frac{1}{J_1}(\mathbf{F}' - \frac{\mathbf{G}'\mathbf{G}'^{\top}H^{-1}\mathbf{F}'}{1/\xi + \mathbf{G}'H^{-1}\mathbf{G}'^{\top}}) & (97) \end{aligned}$$

This last expression can be rewritten using elements of the Fisher information matrix, whose inverse bounds the covariance of any joint estimator of the signal and nuisance variables, $(\hat{s}, \hat{\nu})$:

$$\mathbf{J}(s, \nu) = \begin{bmatrix} J_{11} & J_{12} \\ J_{12} & J_{22} \end{bmatrix} = \begin{bmatrix} \mathbf{F}'^{\top}H^{-1}\mathbf{F}' & \mathbf{F}'^{\top}H^{-1}\mathbf{G}' \\ \mathbf{G}'^{\top}H^{-1}\mathbf{F}' & \mathbf{G}'^{\top}H^{-1}\mathbf{G}' \end{bmatrix} \tag{98}$$

With these substitutions, we have

$$\text{Cov}(\mathbf{R}, \hat{s}|s, \nu) = \frac{1}{J_1}\left(\mathbf{F}' - \frac{J_{12}}{1/\xi + J_{22}}\mathbf{G}'\right) \tag{99}$$

The denominator of Equation 89 involves the variance of the sufficient statistics,

$$\sigma^2_{R_k|s,n} = H_{kk} \tag{100}$$

and the variance of the brain's decoder,

$$\begin{aligned}\sigma^2_{\hat{s}} &= \mathbf{w}^\top H \mathbf{w} \\ &= \mathbf{w}^\top(\Gamma - \xi \mathbf{G}'\mathbf{G}'^\top)\mathbf{w} \\ &= \frac{1}{J_1} - \frac{J_{12}^2}{\xi J_1^2}\frac{1}{(1/\xi + J_{22})^2}\end{aligned} \tag{101}$$

where we used the following results:

$$\begin{aligned}\mathbf{w}^\top \mathbf{G}'\mathbf{G}'^\top \mathbf{w} &= \left(\frac{\mathbf{F}'\Gamma^{-1}}{J_1}\mathbf{G}'\right)^2 \\ &= \frac{1}{J_1^2}\left(\mathbf{F}'H^{-1}\mathbf{G}' - \frac{\mathbf{F}'\Gamma^{-1}\mathbf{G}'\mathbf{G}'H^{-1}\mathbf{G}'}{1/\xi + \mathbf{G}'H^{-1}\mathbf{G}'}\right)^2 \\ &= \frac{1}{J_1^2}\left(J_{12} - \frac{J_{12}J_{22}}{1/\xi + J_{22}}\right)^2 \\ &= \frac{J_{12}^2}{\xi^2 J_1^2}\frac{1}{(1/\xi + J_{22})^2}\end{aligned} \tag{102}$$

Combining the results from Equation 99, 101 and 100, we can compute Equation 89

$$\begin{aligned}c^{\text{sub}}_{R_k} &= \text{Corr}(R_k, \hat{s}|s,\nu) \\ &= \frac{\text{Cov}(R_k, \hat{s}|s,\nu)}{\sigma_{R_k|s,n}\sigma_{\hat{s}|s,n}} \\ &= \frac{\frac{1}{J_1}\left(F'_k - \frac{J_{12}}{1/\xi + J_{22}}G'_k\right)}{\sqrt{H_{kk}}\sigma_{\hat{s}|s,n}}\end{aligned} \tag{103}$$

The optimal choice correlation when there is natural nuisance variation (Eq. 7) is given by

$$C^{\text{opt}}_{R_k} = \sqrt{\frac{J_{1,R_k}}{J_1}} = \frac{F'_k}{\sigma_{R_k|s}\sqrt{J_1}} \tag{104}$$

where $J_{1,R_k} = F'_k/\sigma_{R_k|s}$ is the Fisher Information in R_k about s when there is natural nuisance variation, and $\sigma_{R_k|s} = \sqrt{H_{kk} + \xi G'^2_k}$ is the standard deviation of the statistic R_k, again when there is natural nuisance variation.

The choice correlations for the same decoder differ under experimental conditions with and without nuisance variation: $C^{\text{opt}}_{R_k}$ and $c^{\text{sub}}_{R_k}$. We find that the nuisance-conditioned choice correlations $c^{\text{sub}}_{R_k}$ relate to the optimal nuisance-averaged choice correlations $C^{\text{opt}}_{R_k}$ according to

$$c^{\text{sub}}_{R_k} = \beta_k C^{\text{opt}}_{R_k} - \gamma_k \tag{105}$$

where we have defined the following constants:

$$\begin{aligned}\beta_k &= \frac{\sigma_{R_k|s}}{\sigma_{R_k|s,n}}\frac{1}{\sqrt{J_1}\sigma_{\hat{s}|s,n}} \\ &= \sqrt{\frac{H_{kk} + \xi G'^2_k}{H_{kk}}}\frac{1}{\sqrt{J_1}\sigma_{\hat{s}|s,n}} \\ &= \sqrt{\frac{H_{kk} + \xi G'^2_k}{H_{kk}}}\frac{1}{\sqrt{1 - \frac{J_{12}^2}{\xi J_1}\frac{1}{(1/\xi + J_{22})^2}}},\end{aligned} \tag{106}$$

and

$$\gamma_k = \frac{G'_k}{\sqrt{H_{kk}}}\frac{J_{12}}{(1/\xi + J_2)J_1\sigma_{\hat{s}|s,n}} \tag{107}$$

The slope β_k and offset γ_k of the relationship between these two types of choice correlations (Equation 105) depends on the amount of nuisance variation compared to internal noise and the suboptimality of the brain's decoding strategy. When the signal and nuisance can be disentangled, that is, estimated nearly independently using the statistics $\mathbf{R}(\mathbf{r})$, then J_{12} is small and the choice correlations driven purely by internal fluctuations closely match the optimal choice correlations in the presence of nuisance variation (Figure S2A). In contrast, when nuisance variations remain partialy confused with the signal, then J_{12} is large and the choice correlations for fixed nuisance variables may differ from the optimal pattern seen when allowing nuisance variables to change from trial to trial (Figure S2B).

For the simulations in Figure S2, we set the sufficient statistics to be linear $\mathbf{R}(\mathbf{r}) = \mathbf{r}$ for simplicity. Neural responses were generated from a Gaussian distribution with a stimulus-dependent mean and identity covariance $H = I$: $p(\mathbf{r}|s,\nu) = \mathcal{N}(\mathbf{F}'s + \mathbf{G}'\nu, I)$. In Figure S2A, $\mathbf{F}'$ and $\mathbf{G}'$ are set to be orthogonal to ensure $J_{12} = \mathbf{F}'^\top H^{-1}\mathbf{G}' = 0$. They are picked from the eigenvector of a symmetric matrix $A^\top A$, where A is a matrix whose elements are generated from uniform distribution bounded by 0 and 1. In Figure S2B, each element in $\mathbf{F}'$ and $\mathbf{G}'$ is drawn from a uniform distribution over the interval $[0, 1]$. We simulate 10000 responses of a population with $N = 50$ neurons. The stimulus is set to 0 and the nuisance is fixed to be 1. The brain's decoder assumes a Gaussian prior over the nuisance

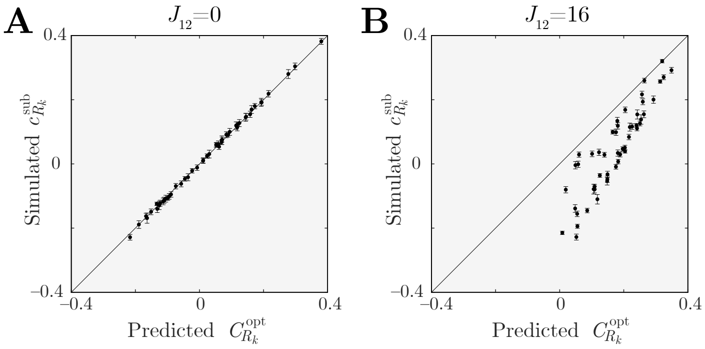

Figure S2: Comparing choice correlations caused by internal and external noise. (**A**) When estimates of nuisance variables are independent of estimates of task-relevant signals, the optimal choice correlations driven by internal noise, $c^{\rm sub}_{R_k}$, match the optimal pattern $C^{\rm opt}_{R_k}$ expected for optimal decoding under natural nuisance variation (Equation 7). (**B**) When the signal and nuisance variables remain confounded by an estimator and decoding is evaluated under different conditions than those for which it was optimized, then the choice correlations need not match this optimal prediction. Means and standard deviations (denoted by error bars) for simulated choice correlations were compute by repeating the tests for 10 times independently.

variation with zero mean and variance $\xi = 2$. The decoding weights follow Equation 86, and the stimulus is estimated using Equation 87. Choice correlations in this fixed-nuisance experiment are computed by Equation 88 (vertical axis in Figure S2). The predicted optimal choice correlation is computed by Equation 104 (horizontal axis in Figure S2). In this setting, $\beta_k \approx 1$ when $J_{12} = 0$.

S.5 Coarse discrimination and choice correlations

We now derive a relationship between nonlinear neural thresholds and nonlinear choice correlations for *coarse* binary discrimination tasks, choosing between stimulus s_+ and s_-. The main ideas are the same as for fine discrimination, but there are a few more subtleties involved when the statistical structure of the response depends on the stimulus.

We assume the brain decodes neural activity $\mathbf{r}$ as a linear weighted sum of nonlinear statistics $\mathbf{R}(\mathbf{r})$, using weights given by linear regression as

$$\mathbf{w} \propto \mathrm{Cov}(\mathbf{R})^{-1}\mathrm{Cov}(\mathbf{R}, s) \tag{108}$$

The latter factor reflects the signal strength,

$$\begin{aligned}\mathrm{Cov}(\mathbf{R}, s) &= \langle \mathbf{R}s\rangle - \langle \mathbf{R}\rangle\langle s\rangle &(109)\\ &= \tfrac{1}{2}(\mathbf{F}_+ - \mathbf{F}_-)ds = \tfrac{1}{2}\Delta\mathbf{F}\, ds &(110)\end{aligned}$$

We assume that the two values $s_\pm = s_0 \pm \Delta s$ are equally probable, and notate the mean responses as $\mathbf{F}_\pm = \mathbf{F}(s_\pm) = \langle \mathbf{R}|s_\pm\rangle$. The factor $\mathrm{Cov}\,\mathbf{R}$ includes covariability induced by both signal and noise. Using the law of total covariance, these contributions can be separated as

$$\begin{aligned}\mathrm{Cov}\,\mathbf{R} &= \langle \mathrm{Cov}(\mathbf{R}|s)\rangle_s + \mathrm{Cov}\,\langle \mathbf{R}|s\rangle &(111)\\ &= \bar{\Gamma} + \tfrac{1}{4}\Delta\mathbf{F}\Delta\mathbf{F}^\top &(112)\end{aligned}$$

where the first term is the average noise covariance across the stimulus ensemble, $\bar{\Gamma} = \langle \mathrm{Cov}(\mathbf{R}|s)\rangle_s$, and the second term reflects variance along the signal direction. As for fine discrimination, noise variance along the signal direction has no influence on the optimal readout direction, since it cannot be removed. Using the Sherman-Morrison formula, we find that the decoder is

$$\begin{aligned}\mathbf{w} &\propto \mathrm{Cov}(\mathbf{R})^{-1}\,\mathrm{Cov}(\mathbf{R}, s)\\ &= \left(\bar{\Gamma} + \tfrac{1}{4}\Delta\mathbf{F}\Delta\mathbf{F}^\top\right)^{-1} \tfrac{1}{2}\Delta\mathbf{F}\,\Delta s\\ &\propto \left(\bar{\Gamma}^{-1} - \frac{\frac{1}{4}\bar{\Gamma}^{-1}\Delta\mathbf{F}\Delta\mathbf{F}^\top\bar{\Gamma}^{-1}}{1 + \frac{1}{4}\Delta\mathbf{F}^\top\bar{\Gamma}^{-1}\Delta\mathbf{F}}\right)\tfrac{1}{2}\Delta\mathbf{F}\\ &\propto \bar{\Gamma}^{-1}\Delta\mathbf{F} &(113)\end{aligned}$$

For unbiased decoding, the proportionality is given by $1/\Delta\mathbf{F}^\top\bar{\Gamma}^{-1}\Delta\mathbf{F}$.

S.5.1 Average conditional choice correlations

The core desideratum for a measure of choice correlations is to isolate the non-stimulus fluctuations that correlate with choices. The typical way to ensure this is to measure correlations between neural responses and choices only when the stimulus is completely ambiguous, *i.e.* at the decision boundary. Other studies have sought to expand the range of stimuli that can be used for these correlations [10, 13]. Mathematically, we examine the statistical relationship between neural responses and choices that remains after *conditioning* on the stimulus, via $p(\mathbf{R}, \hat{s}|s)$. Here we quantify this relationship through a conditional covariance, $\mathrm{Cov}(\mathbf{R}, \hat{s}|s)$. For coarse discrimination, the strength (and pattern) of this correlation may depend on the particular stimulus used. To account for this, we compute an average over possible stimuli, $\langle \mathrm{Cov}(R_k, \hat{s}|s)\rangle_s$. If we normalize by root mean variances, we obtain

$$B_{R_k} = \frac{\langle \mathrm{Cov}(R_k, \hat{s}|s)\rangle_s}{\sqrt{\langle \mathrm{Var}(R_k|s)\rangle_s\,\langle \mathrm{Var}(\hat{s}|s)\rangle_s}} \tag{114}$$

This nonlinear choice correlation can be rewritten as

$$\begin{aligned} B_{R_k} &= \frac{(\mathbf{w}^\top \langle \text{Cov}(\mathbf{R}|s)\rangle_s)_k}{\sqrt{\bar{\Gamma}_{kk}\ \mathbf{w}^\top \langle \text{Cov}(\mathbf{R}|s)\rangle_s \mathbf{w}}} \\ &= \frac{(\Delta\mathbf{F}^\top\bar{\Gamma}^{-1}\bar{\Gamma})_k}{\sqrt{\bar{\Gamma}_{kk}\ \Delta\mathbf{F}^\top\bar{\Gamma}^{-1}\bar{\Gamma}\bar{\Gamma}^{-1}\Delta\mathbf{F}}} \\ &= \frac{\Delta\mathbf{F}_k}{\sqrt{\bar{\Gamma}_{kk}\ \Delta\mathbf{F}^\top\bar{\Gamma}^{-1}\Delta\mathbf{F}}} \end{aligned} \tag{115}$$

We recognize that this expression contains the ratio of sensitivities for the neural statistic R_k and the entire population $\mathbf{r}$ in coarse discrimination, $d'_k = \Delta\mathbf{F}_k/\sqrt{\bar{\Gamma}_{kk}}$ and $d' = \sqrt{\Delta\mathbf{F}^\top\bar{\Gamma}^{-1}\Delta\mathbf{F}}$. We therefore find the same result as for optimal fine discrimination (Eq. 18):

$$B^{\text{opt}}_{R_k} = \frac{d'_k}{d'} \tag{116}$$

S.5.2 Signal estimation from total correlations

It is useful to express the discriminability through the total correlation between the responses and the stimulus,

$$D_{R_k,s} = \text{Corr}(R_k, s) \tag{117}$$

$$\begin{aligned} &= \frac{\text{Cov}(R_k, s)}{\sigma_{R_k}\sigma_s} \\ &= \frac{\frac{1}{2}\Delta F_k\, ds}{\sqrt{(\bar{\Gamma}_{kk} + \frac{1}{4}\Delta F_k^2)\sigma_s^2}} \\ &= \frac{1}{\sqrt{\frac{4\bar{\Gamma}_{kk}}{\Delta F_k^2} + 1}} \\ &= \frac{1}{\sqrt{4{d'_k}^{-2} + 1}} \end{aligned} \tag{118}$$

In these equations we used the fact that for binary discrimination, the standard deviation of the signal is related to the difference between the two possible signal values, $\sigma_s = \frac{1}{2}(s_+ - s_-) = ds$. We can invert Eq. 118 to find

$$d'_k = \frac{2}{\sqrt{D_{R_k,s}^{-2} - 1}} \tag{119}$$

This dependence is plotted in Figure S3.

Similarly, we can express the behavioral discriminability d' in terms of the correlation between the estimate and the stimulus, $D_{\hat{s},s} = \text{Corr}(\hat{s}, s)$:

$$d' = \frac{2}{\sqrt{D_{\hat{s},s}^{-2} - 1}} \tag{120}$$

The relationship between discriminability d' and total correlation D is linear when D is relatively small. Thus we can approximate the optimal nonlinear choice correlation as:

$$B^{\text{opt}}_{R_k} \approx \frac{D_{R,s}}{D_{\hat{s},s}} \tag{121}$$

Here the total correlation is computed based on a continuous estimate $\hat{s}$. When the behavioral outcome is a binary choice, this relationship is more complicated. Section S.6.3 calculates the relationship between $D_{\hat{s}}$ and $D_{\hat{s}_\pm}$ for one particular task.

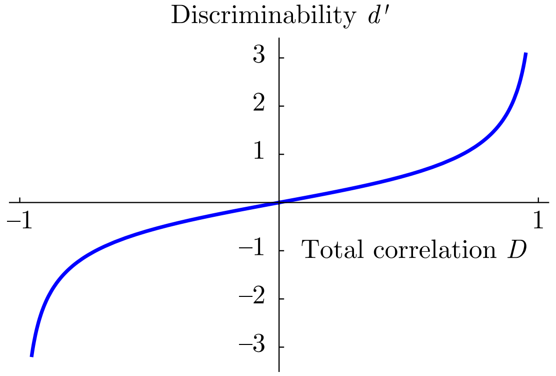

Figure S3: Stimulus discriminability d' for a response variable R, versus total correlation between that variable and the stimulus, $D = \text{Corr}\,(R, s)$, according to Eq. 119.

S.6 Orientation variance discrimination task

S.6.1 Coarse tasks: Continuous estimation versus binary discrimination

The experiment of Section **Evidence for optimal nonlinear computation in macaque brains** defines an orientation variance discrimination task in which the relevant statistics are quadratic functions of the orientation. The quadratic decoding model described in the main text could suffice for this problem. However, in our case the variances to be distinguished are quite different, such that the nuisance variation differs substantially between these two stimulus categories. As described in Methods **Optimality test**, coarse tasks with stimulus-dependent variability generate a slightly different prediction compared to fine tasks (or coarse tasks with stimulus-independent variability).

Moreover, there are minor differences between the predictions for continuous estimation and binary dis-

crimination, and these differences are more complicated for coarse tasks than fine ones. Here we describe in detail the somewhat lengthy computation of the ratio ζ between choice correlations for continuous quadratic estimation and binary quadratic decoding. For coarse discrimination, the ratio ζ will depend on the input statistics and threshold, but for fine discrimination ζ becomes a constant. Regardless, for our cases of interest these numbers are generally near 1.

We begin by assuming that the variance estimate is the square of the orientation estimate $\hat{s} = \hat{\phi}^2$, and a binary guess about the variance is given by $\hat{s}_\pm = \mathrm{sgn}\,(\hat{\phi}^2 - \theta^2)$ where θ is the animal's orientation threshold. We assume that $\hat{\phi}$ is an unbiased estimate of the orientation ϕ, so $\langle \hat{\phi}|\phi\rangle = \phi$. We denote one neuron's mean response to the orientation by $\langle r|\phi\rangle = \mu(\phi)$ which we approximate linearly as $\mu(\phi) \approx \bar{\mu} + \mu'\phi$ with $\bar{\mu} = \mu(0)$. The mean behavioral choice is $\langle \hat{s}_\pm|s\rangle = m_s$. Since the stimulus is binary, we will denote this mean with a subscript, $\langle \hat{s}_\pm|s_+\rangle = m_+$ or $\langle \hat{s}_\pm|s_-\rangle = m_-$.

The joint distribution $p(r, \hat{\phi}|s)$ arises from both internal noise and nuisance variation, $p(r, \hat{\phi}|s) = \int d\phi\, p(r, \hat{\phi}|\phi)p(\phi|s)$. For a given orientation ϕ, the neural response r and orientation estimate $\hat{\phi}$ follow a bivariate normal distribution,

$$p(r, \hat{\phi}|\phi) = \mathcal{N}\left(\begin{matrix} r \\ \hat{\phi}\end{matrix}\middle| \begin{matrix}\mu(\phi) \\ \phi\end{matrix}; \begin{bmatrix} H_{rr|\phi} & H_{r\hat{\phi}|\phi} \\ H_{r\hat{\phi}|\phi} & H_{\hat{\phi}\hat{\phi}|\phi}\end{bmatrix}\right) \tag{122}$$

which summarizes all of the internal noise given the sensory input.

By design, the orientation variable ϕ is driven by a normally distributed nuisance variable ν, with $\phi = \sqrt{s}\nu$ and $p(\phi|s) = \mathcal{N}(\phi|0, s)$, so we can write the marginal distribution $p(r, \hat{\phi}|s)$ as

$$\begin{aligned} p(r, \hat{\phi}|s) &= \mathcal{N}\left(\begin{matrix} r \\ \hat{\phi}\end{matrix}\middle| \begin{matrix}\bar{\mu} \\ 0\end{matrix}; \begin{bmatrix} H_{rr|\phi} + \mu'^2 s & H_{r\hat{\phi}|\phi} + \mu' s \\ H_{r\hat{\phi}|\phi} + \mu' s & H_{\hat{\phi}\hat{\phi}|\phi} + s\end{bmatrix}\right) \\ &= \mathcal{N}(\mu(s), \Sigma(s)) \end{aligned} \tag{123}$$

For now we suppress the explicit dependence on s.

The conditional covariance between the nonlinear statistic R and choice is

$$\begin{aligned} &\mathrm{Cov}(\hat{s}_\pm, R|s) \\ &= \left\langle \mathrm{sgn}\,(\hat{\phi}^2 - \theta^2) r^2 \right\rangle_{r,\hat{\phi}} - \left\langle \mathrm{sgn}\,(\hat{\phi}^2 - \theta^2)\right\rangle_{\hat{\phi}} \left\langle r^2\right\rangle_r \end{aligned} \tag{124}$$

where $R = r^2$ and we reiterate that we are suppressing the conditioning on s. The second moment is

$$\begin{aligned} &\left\langle \mathrm{sgn}\,(\hat{\phi}^2 - \theta^2) r^2 \right\rangle_{r,\hat{\phi}} \\ &= \left\langle \mathrm{sgn}\,(\hat{\phi}^2 - \theta^2) \left\langle r^2|\hat{\phi}\right\rangle_{r|\hat{\phi}} \right\rangle_{\hat{\phi}} \\ &= \left\langle \mathrm{sgn}\,(\hat{\phi}^2 - \theta^2)(\Sigma_{r|\hat{\phi}} + \mu^2_{r|\hat{\phi}}) \right\rangle_{\hat{\phi}} \qquad (125) \\ &= \left\langle \mathrm{sgn}\,(\hat{\phi}^2 - \theta^2) \left[\Sigma_{rr} - \frac{\Sigma^2_{r\hat{\phi}}}{\Sigma_{\hat{\phi}\hat{\phi}}} + \left(\mu + \frac{\Sigma_{r\hat{\phi}}}{\Sigma_{\hat{\phi}\hat{\phi}}}\hat{\phi}\right)^2\right] \right\rangle_{\hat{\phi}} \end{aligned}$$

where we used the conditional distribution

$$p(r|\hat{\phi}) = \mathcal{N}\left(r \middle| \mu + \frac{\Sigma_{r\hat{\phi}}}{\Sigma_{\hat{\phi}\hat{\phi}}}\hat{\phi},\, \Sigma_{rr} - \frac{\Sigma^2_{r\hat{\phi}}}{\Sigma_{\hat{\phi}\hat{\phi}}}\right) \tag{126}$$

This can be written as

$$\left\langle \mathrm{sgn}\,(\hat{\phi}^2 - \theta^2)(a\hat{\phi}^2 + b\hat{\phi} + c)\right\rangle_{\hat{\phi}} \tag{127}$$

for coefficients

$$a = \frac{\Sigma^2_{r\hat{\phi}}}{\Sigma^2_{\hat{\phi}\hat{\phi}}} \tag{128}$$

$$b = 2\frac{\Sigma_{r\hat{\phi}}}{\Sigma_{\hat{\phi}\hat{\phi}}}\mu \tag{129}$$

$$c = \Sigma_{rr} - \frac{\Sigma^2_{r\hat{\phi}}}{\Sigma_{\hat{\phi}\hat{\phi}}} + \mu^2 \tag{130}$$

Note that this is an expectation over $\hat{\phi}$ only. Such an expected value can be written as a sum of integrals:

$$\begin{aligned} &\left\langle \mathrm{sgn}\,(\hat{\phi}^2 - \theta^2)\hat{\phi}^\alpha\right\rangle_{\hat{\phi}} \\ &= \left[\int_{-\infty}^{-\theta} - \int_{-\theta}^{\theta} + \int_{\theta}^{\infty}\right] \hat{\phi}^\alpha p(\hat{\phi}) d\hat{\phi} \\ &= \left[\int_{-\infty}^{-\theta} - \left(\int_{-\infty}^{\theta} - \int_{-\infty}^{-\theta}\right) + \left(\int_{-\infty}^{\infty} - \int_{-\infty}^{\theta}\right)\right] \hat{\phi}^\alpha p(\hat{\phi}) d\hat{\phi} \\ &= \left[2\int_{-\infty}^{-\theta} - 2\int_{-\infty}^{\theta} + \int_{-\infty}^{\infty}\right] \hat{\phi}^\alpha p(\hat{\phi}) d\hat{\phi} \end{aligned} \tag{131}$$

These integrals can be expressed in terms of error functions, where $\sigma^2_{\hat{\phi}}$ is the marginal variance for $p(\hat{\phi}|s)$:

$$\int_{-\infty}^{\theta} d\hat{\phi}\, \hat{\phi}^0 \mathcal{N}(\hat{\phi}|0, \sigma^2_{\hat{\phi}}) = \tfrac{1}{2}\,\mathrm{erfc}\left(\tfrac{\theta}{\sqrt{2}\sigma_{\hat{\phi}}}\right) \tag{132}$$

$$\int_{-\infty}^{\theta} d\hat{\phi}\, \hat{\phi}^1 \mathcal{N}(\hat{\phi}|0, \sigma^2_{\hat{\phi}}) = -p_{\hat{\phi}}(\theta)\sigma^2_{\hat{\phi}} \tag{133}$$

$$\int_{-\infty}^{\theta} d\hat{\phi}\, \hat{\phi}^2 \mathcal{N}(\hat{\phi}|0, \sigma^2_{\hat{\phi}}) = \tfrac{1}{2}\,\mathrm{erfc}\left(\tfrac{\theta}{\sqrt{2}\sigma_{\hat{\phi}}}\right)\sigma^2_{\hat{\phi}} - p_{\hat{\phi}}(\theta)\sigma^2_{\hat{\phi}}\theta \tag{134}$$

Note that $p_{\hat{\phi}}(\theta)$ has units of $[\phi]^{-1}$, so units are consistent across these expressions.

Combining these with Eq. 131 we obtain

$$m = \left\langle \operatorname{sgn}(\hat{\phi}^2 - \theta^2) \right\rangle = 2\operatorname{erfc}\left(\frac{\theta}{\sqrt{2}\sigma}\right) - 1 \tag{135}$$

$$\left\langle \operatorname{sgn}(\hat{\phi}^2 - \theta^2)\hat{\phi} \right\rangle = 0 \tag{136}$$

$$\left\langle \operatorname{sgn}(\hat{\phi}^2 - \theta^2)\hat{\phi}^2 \right\rangle = \sigma^2_{\hat{\phi}} m + 4\theta\sigma^2_{\hat{\phi}} p_{\hat{\phi}}(\theta) \tag{137}$$

where we have used the identity $\operatorname{erfc}(-x) = 2 - \operatorname{erfc}(x)$ and the symmetry $p_{\hat{\phi}}(\theta) = p_{\hat{\phi}}(-\theta)$. The first term, m, is the mean of $\hat{s}_\pm$, and will appear several times in the equations below.

Returning to Eq. 125, we have

$$\begin{aligned}
&\operatorname{Cov}(\hat{s}_\pm, R|s) \\
&= \left\langle \operatorname{sgn}(\hat{\phi}^2 - \theta^2) r^2 \right\rangle_{r,\hat{\phi}} - \left\langle \operatorname{sgn}(\hat{\phi}^2 - \theta^2) \right\rangle_{\hat{\phi}|s} \left\langle r^2 \right\rangle_r \\
&= \left\langle \operatorname{sgn}(\hat{\phi}^2 - \theta^2)(a\hat{\phi}^2 + b\hat{\phi} + c) \right\rangle_{\hat{\phi}} \\
&\quad - \left\langle \operatorname{sgn}(\hat{\phi}^2 - \theta^2) \right\rangle_{\hat{\phi}} \left\langle r^2 \right\rangle_r \\
&= a\left[\sigma^2_{\hat{\phi}} m + 4\theta\sigma^2_{\hat{\phi}} p_{\hat{\phi}}(\theta)\right] m + cm - m(\Sigma_{rr} + \mu^2) \\
&= 4\theta \frac{\Sigma^2_{r\hat{\phi}}}{\Sigma_{\hat{\phi}\hat{\phi}}} p_{\hat{\phi}}(\theta)
\end{aligned} \tag{138}$$

Note that all of the erfc terms have canceled.

Compare that to the corresponding covariance for continuous estimation,

$$\operatorname{Cov}(\hat{s}, R|s) = 2\Sigma^2_{r\hat{\phi}} \tag{139}$$

The conditional variance of a binary output $\hat{s} = \pm 1$ is simply

$$\operatorname{Var}(\hat{s}_\pm|s) = 1 - \langle \hat{s}_\pm|s \rangle^2 \tag{140}$$

$$= 1 - m^2 \tag{141}$$

whereas, the variance for the continuous estimator is

$$\operatorname{Var}(\hat{s}|s) = \left\langle (\hat{\phi}^2 - \theta^2)^2 |s \right\rangle - \left\langle \hat{\phi}^2 - \theta^2 |s \right\rangle^2 \tag{142}$$

$$= 2\Sigma^2_{\hat{\phi}\hat{\phi}} \tag{143}$$

The variance of r^2, $\operatorname{Var}(r^2|s) = 2\Sigma^2_{rr} + 4\Sigma_{rr}\mu^2$, is the same whether the behavioral estimate is continuous or binary.

Our goal here is to compute the change in our measure of nonlinear choice correlation, namely,

$$\zeta = \frac{B^\pm_R}{B_R} = \frac{\frac{\langle \operatorname{Cov}(\hat{s}_\pm, R|s)\rangle_s}{\sqrt{\langle \operatorname{Var}(\hat{s}_\pm|s)\rangle_s \langle \operatorname{Var}(R|s)\rangle_s}}}{\frac{\langle \operatorname{Cov}(\hat{s}, R|s)\rangle_s}{\sqrt{\langle \operatorname{Var}(\hat{s}|s)\rangle_s \langle \operatorname{Var}(R|s)\rangle_s}}} \tag{144}$$

where the averages over $p(s) = 1/2$ include equal proportions of the binary stimuli s_+ and s_-. Substituting our calculations above, and reintroducing the dependencies on s, we find

$$\zeta = \frac{\langle \operatorname{Cov}(\hat{s}_\pm, R|s)\rangle_s}{\langle \operatorname{Cov}(\hat{s}, R|s)\rangle_s} \sqrt{\frac{\langle \operatorname{Var}(\hat{s}|s)\rangle_s}{\langle \operatorname{Var}(\hat{s}_\pm|s)\rangle_s}} \tag{145}$$

$$= \frac{\sum_s p(s) 4\theta \frac{\Sigma^2_{r\hat{\phi}|s}}{\Sigma_{\hat{\phi}\hat{\phi}|s}} p_{\hat{\phi}}(\theta|s)}{\sum_s p(s) 2\Sigma^2_{r\hat{\phi}|s}} \sqrt{\frac{\sum_s p(s) 2\Sigma^2_{\hat{\phi}\hat{\phi}|s}}{\sum_s p(s)(1 - m_s^2)}} \tag{146}$$

For tasks where the variability is dominated by external nuisance variables rather than by internal noise, i.e. $H \ll s(\mu', 1)(\mu', 1)^\top$, we can approximate the covariances by $\Sigma_{rr} \approx \mu'^2 s$, $\Sigma_{r\hat{\phi}} \approx \mu' s$, and $\Sigma_{\hat{\phi}\hat{\phi}} \approx s$. Substituting these approximations into the expression above, we obtain

$$\zeta \approx \frac{\frac{1}{2}\sum_s 4\theta \frac{\mu'^2 s^2}{s} \frac{e^{-\theta^2/2s}}{\sqrt{2\pi s}}}{\frac{1}{2}\sum_s 2\mu'^2 s^2} \sqrt{\frac{\frac{1}{2}\sum_s 2s^2}{1 - \frac{1}{2}\sum_s m_s^2}} \tag{147}$$

In our task conditions, $3 = \sqrt{s_-} \ll \sqrt{s_+} = 15$, so some terms dominate in the sums. Moreover, we assume that the threshold θ lies far enough between $\sqrt{s_-} < \theta < \sqrt{s_+}$ that $e^{-\theta^2/2s_-} \approx 0$ and $e^{-\theta^2/2s_+} \approx 1$. We then find

$$\zeta \approx \frac{\frac{1}{2} 4\theta \sqrt{\frac{s_+}{2\pi}}}{\frac{1}{2} 2 s_+^2} \sqrt{\frac{\frac{1}{2} 2 s_+^2}{1 - \frac{1}{2}\sum_s m_s^2}} \tag{148}$$

$$= \frac{2}{\sqrt{\pi}} \frac{\theta}{\sqrt{s_+}} \frac{1}{\sqrt{1 - \frac{1}{2}\sum_s m_s^2}} \tag{149}$$

Empirically, we find that $1 - \langle m^2 \rangle_s \approx \frac{1}{2}$ (Figure S4). In that case, we obtain

$$\zeta \approx \frac{2\theta}{\sqrt{\pi s_+}} \tag{150}$$

This expression is independent of the statistics of r. Therefore the same correction factor holds for cross-terms like $r_j r_k$, which can be expressed as linear combination of squares, $R_{jk} = r_j r_k = \frac{1}{2}(r_j + r_k)^2 - \frac{1}{2}r_j^2 - \frac{1}{2}r_k^2$. We use this correction factor ζ to adjust our predicted quadratic choice correlations in Figure 6.

To find the behavioral threshold θ for Eq. 150, we used logistic regression of choice $\hat{s}_\pm$ on the absolute value of the stimulus orientation, $|\phi|$, and assign the threshold θ to be the orientation where the probability of both choices was equal.

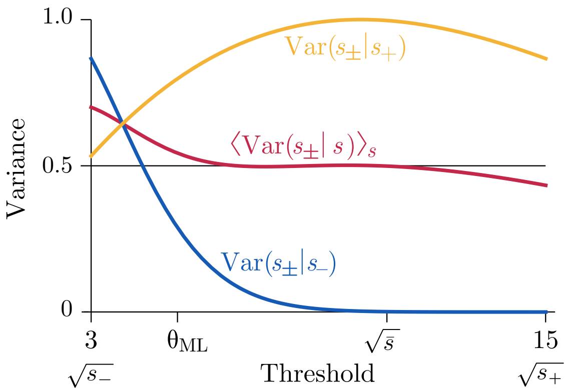

Figure S4: The average variance of $\hat{s}_\pm$ conditioned on the stimulus s (red) is approximately 1/2 over a wide range of thresholds.

S.6.2 Fine tasks: Continuous estimation versus binary discrimination

For fine discrimination, the stimulus s is effectively constant, so we need not take averages.

$$\zeta^{\text{fine}} = \frac{\text{Cov}\left(\hat{s}_\pm, R|s\right)}{\text{Cov}\left(\hat{s}, R|s\right)}\sqrt{\frac{\text{Var}\left(\hat{s}|s\right)}{\text{Var}\left(\hat{s}_\pm|s\right)}} \tag{151}$$

$$= \frac{4\theta\frac{\Sigma^2_{r\hat{\phi}|s}}{\Sigma_{\hat{\phi}\hat{\phi}|s}}p_{\hat{\phi}}(\theta|s)}{2\Sigma^2_{r\hat{\phi}|s}}\sqrt{\frac{2\Sigma^2_{\hat{\phi}\hat{\phi}|s}}{1-m_s^2}} \tag{152}$$

After several cancelations, and using the fact that for fine discrimination, $\theta = s \approx s_+ \approx s_-$, we find

$$\zeta^{\text{fine}} = \frac{e^{-\frac{1}{2}}}{\sqrt{2\pi}}\sqrt{\frac{8}{1-(2\,\text{erfc}(\frac{1}{\sqrt{2}})-1)^2}} \tag{153}$$

$$\approx 0.735 \tag{154}$$

Observe that for fine discrimination, the ratio ζ is a constant, independent of the underlying statistics.

S.6.3 Total correlation for binary and continuous estimates

We showed in Methods **Optimality test** that the discriminability is related to the total correlation between signal and response. However, those relationships were based on continuous estimates of the binary stimulus. As above, when the behavioral choice is also binary, we can adjust the calculation slightly. Here we compare the total correlations for continuous and binary response, $D_{\hat{s},s}$ and $D_{\hat{s}_\pm,s}$.

$$\begin{aligned}
\text{Cov}\left(\hat{s}, s\right) &= \left\langle\langle\hat{s}|s\rangle\, s\right\rangle_s - \langle\hat{s}\rangle\,\langle s\rangle \\
&= \left\langle\left\langle\hat{\phi}^2|s\right\rangle s\right\rangle_s - \left\langle\left\langle\hat{\phi}^2|s\right\rangle\right\rangle_s \bar{s} \\
&= \left\langle\Sigma_{\hat{\phi}\hat{\phi}|s}s\right\rangle_s - \left\langle\left\langle\Sigma_{\hat{\phi}\hat{\phi}}|s\right\rangle\right\rangle_s \bar{s} \\
&= \left\langle(H_{\hat{\phi}\hat{\phi}}+s)s\right\rangle_s - \left\langle(H_{\hat{\phi}\hat{\phi}}+s)\right\rangle_s \bar{s} \\
&= H_{\hat{\phi}\hat{\phi}}\bar{s} + \left\langle s^2\right\rangle_s - (H_{\hat{\phi}\hat{\phi}}+\bar{s})\bar{s} \\
&= \text{Var}(s) \\
&= \tfrac{1}{2}(s_+^2+s_-^2) - \tfrac{1}{4}(s_++s_-)^2 \\
&= \tfrac{1}{4}\Delta s^2
\end{aligned} \tag{155}$$

In contrast, the total covariance of $\hat{s}_\pm$ is

$$\begin{aligned}
\text{Cov}\left(\hat{s}_\pm, s\right) &= \left\langle\langle\hat{s}_\pm|s\rangle\, s\right\rangle_s - \langle\hat{s}_\pm\rangle\,\langle s\rangle \\
&= \langle m_s s\rangle_s - \langle m_s\rangle_s\,\bar{s} \\
&= \tfrac{1}{2}\left(m(s_+)s_+ + m(s_-)s_-\right) \\
&\quad - \tfrac{1}{4}\left(m(s_+)+m(s_-)\right)\bar{s} \\
&= \tfrac{1}{4}\Delta m\Delta s
\end{aligned} \tag{156}$$

The total variance of $\hat{s}$ is

$$\begin{aligned}
\text{Var}\left(\hat{s}\right) &= \left\langle\hat{\phi}^4\right\rangle - \left\langle\hat{\phi}^2\right\rangle^2 \\
&= \left\langle\left\langle\hat{\phi}^4|s\right\rangle\right\rangle_s - \left\langle\left\langle\hat{\phi}^2|s\right\rangle\right\rangle_s^2 \\
&= \left\langle 3\Sigma^2_{\hat{\phi}\hat{\phi}|s}\right\rangle_s - \left\langle\Sigma_{\hat{\phi}\hat{\phi}|s}\right\rangle_s^2 \\
&= \tfrac{3}{2}\left(\Sigma^2_{\hat{\phi}\hat{\phi}|+} + \Sigma^2_{\hat{\phi}\hat{\phi}|-}\right) - \left(\tfrac{1}{2}\left(\Sigma_{\hat{\phi}\hat{\phi}|+}+\Sigma_{\hat{\phi}\hat{\phi}|-}\right)\right)^2 \\
&= \tfrac{1}{4}\left(5\Sigma^2_{\hat{\phi}\hat{\phi}|+} - 2\Sigma_{\hat{\phi}\hat{\phi}|+}\Sigma_{\hat{\phi}\hat{\phi}|-} + 5\Sigma^2_{\hat{\phi}\hat{\phi}|-}\right) \\
&= \tfrac{1}{4}(5(H_{\hat{\phi}\hat{\phi}}+s_+)^2 - 2(H_{\hat{\phi}\hat{\phi}}+s_+)(H_{\hat{\phi}\hat{\phi}}+s_-) \\
&\quad + 5(H_{\hat{\phi}\hat{\phi}}+s_-)^2) \\
&= \tfrac{1}{4}(8H^2_{\hat{\phi}\hat{\phi}} + H_{\hat{\phi}\hat{\phi}}(10s_+ - 2s_+ - 2s_- + 10s_-) \\
&\quad + 5s_+^2 - 2s_+s_- + 5s_-^2) \\
&= 2H^2_{\hat{\phi}\hat{\phi}} + 2H_{\hat{\phi}\hat{\phi}}(s_++s_-) \\
&\quad + \tfrac{1}{4}\left(5s_+^2 - 2s_+s_- + 5s_-^2\right)
\end{aligned} \tag{157}$$

In the limit where the nuisance variability dominates the internal variability, and $s_+ \gg s_-$, this simplifies to

$$\text{Var}\left(\hat{s}\right) \approx \tfrac{5}{4}s_+^2 \tag{158}$$

The total variance of $\hat{s}_\pm$ is

$$\text{Var}\left(\hat{s}_\pm\right) = 1 - \langle\hat{s}_\pm\rangle^2 = 1 - \bar{m}^2 \tag{159}$$

where $\bar{m} = \frac{1}{2}(m_+ + m_-)$.

Combining these computations, we see that ratio of total correlations for binary $\hat{s}_\pm$ and continuous $\hat{s}$ is

$$\begin{aligned}\delta &= \frac{D_{\hat{s},s}}{D_{\hat{s}_\pm,s}} \\ &= \frac{\mathrm{Corr}(\hat{s}_\pm, s)}{\mathrm{Corr}\,(\hat{s}, s)} \\ &= \frac{\mathrm{Cov}(\hat{s}_\pm, s)}{\mathrm{Cov}\,(\hat{s}, s)} \sqrt{\frac{\mathrm{Var}\,(\hat{s})}{\mathrm{Var}\,(\hat{s}_\pm)}} \\ &\approx \sqrt{\tfrac{5}{4}} \frac{1}{1 - \frac{s_-}{s_+}} \frac{\Delta m}{\sqrt{1 - \bar{m}^2}} \end{aligned} \tag{160}$$

All of these quantities are measurable from data or are given by the task.

S.6.4 Optimal binary nonlinear coarse choice correlations

We can now combine our results above to create a prediction for optimal binary nonlinear coarse choice correlations. From Eq. 121 and Eq. 144, we have

$$B_R^{\mathrm{opt},\pm} = \zeta \frac{D_{R,s}}{D_{\hat{s},s}} \tag{161}$$

From Eq. 160 we can adjust the

$$D_{\hat{s},s} = \delta D_{\hat{s}_\pm,s} \tag{162}$$

Combining these we have

$$B_R^{\mathrm{opt},\pm} = \frac{\zeta}{\delta} \frac{D_{R,s}}{D_{\hat{s}_\pm,s}} \tag{163}$$

where ζ and δ are determined by experimentally measurable quantities. Their precise values depends on the monkey and the session, but the ratio is typically $\zeta/\delta \approx 0.62 \pm 0.33$. When plotting the data in Figure 6, we apply these corrections to each session before combining different sessions together.

S.6.5 Nonlinear information in internal noise

S.7 Practical considerations in nonlinear choice correlation tests

S.7.1 Information and decoding efficiency in a redundant code

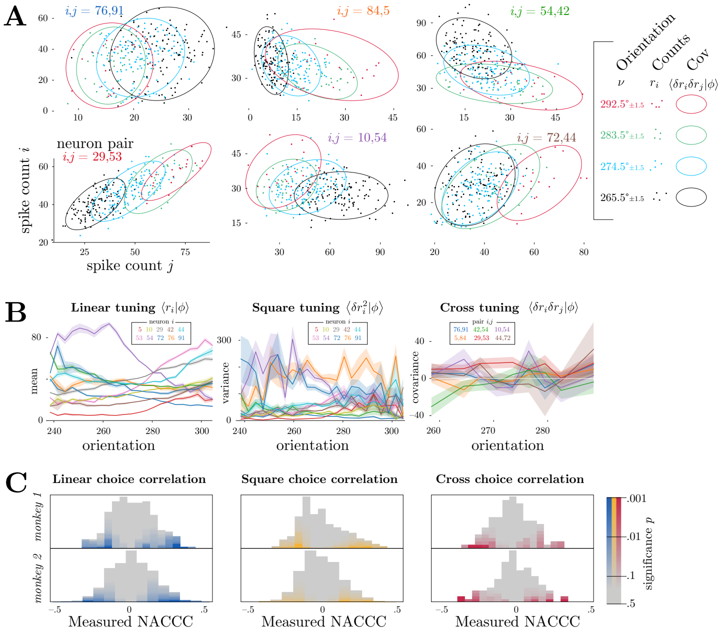

Figure S5: Internal noise covariance is only weakly tuned to orientation and insignificantly tuned to choice. **A**: Scatter plots of neural responses to multiple groups of trials, each group with nearly identical orientations (± 1.5 deg). There are significant shifts in the means and variances of these response clouds, but changes in the correlations are not reliable. Cell pairs with strongest joint quadratic tuning were selected. **B**: For these same selected neurons, direct plots of linear and quadratic tunings confirm that the mean response is strongly tuned to orientation, the response variance is moderately tuned, and the internal noise cross-covariance is not tuned. Solid lines denote mean and shades denote standard deviations of each neural statistics. **C**: Histograms of choice correlations show that purely internal noise is not significantly correlated with choices ($p < 0.01$, two-sample Kolmogorov-Smirnov test for a choice-shuffled null distribution), unlike the nuisance-generated fluctuations seen in Figure 6D. To isolate the correlation of internal noise on choice, we compute the Normalized Average Conditional Choice Correlation (NACCC) where we condition on, and then average over, the *complete* stimulus (s, ν) rather than just on the task-relevant stimulus s as in Eq. 17. Individual choice correlations within the histograms are each colored by their significance according to their own null distribution (Methods **Application to neural data**).

When information is broadly distributed amongst many neurons, the contributions of individual neural responses or functions of those responses could be too small to measure. This potential problem is magnified for higher-order statistics, since there are so many of them. In particular, if there are N neurons in a population, there are $O(N^z)$ of them for a z-th order polynomial nonlinearity. However, for redundant codes, we show that vastly fewer higher-order statistics are required to capture the information.

Consider a redundant code arising from a linear cortical expansion [11]. When a large cortical population of size N inherits all of its information from a smaller upstream sensory neural population of size M, there will be significant redundancy in the recorded cortical neural population. For information contained in z-th order polynomial statistics, there will be only $\sim M^z$ degrees of freedom, which is much smaller than $\sim N^z$.

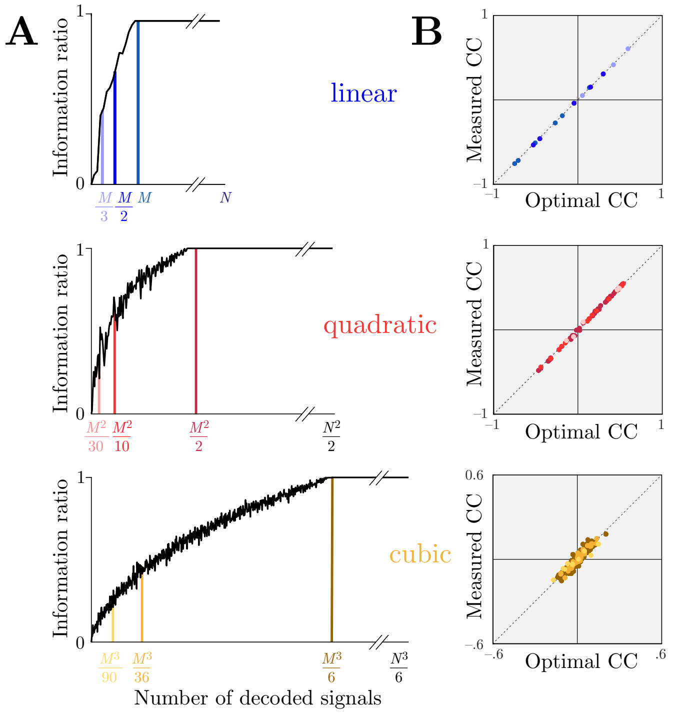

Figure S6: Consequences of redundancy on information and choice correlations. (**A**) Information decoded from various numbers of linear (blue), quadratic (red), and cubic (green) statistics of downstream neural population. Information saturates after decoding $\sim M^z$ units where M is the upstream population size and z is the order of the statistic. (**B**) Nonlinear choice correlations for recordings from a simulated optimal decoder under the same conditions. The brain is assumed to decode optimally while the experimenter only examines a subset of statistics. The slope of 1 predicted for optimal decoding is evident even when recording few informative neural statistics.

Figure S6 shows simulations of a cortical expansion from a smaller population of upstream neurons ($M = 15$) to a much larger population recorded downstream ($N = 100$), with 50000 trials. The upstream neural responses $\rho \in \mathbb{R}^M$ are first generated from cubic codes (Supplemental Materials S.1.4) with third-order sufficient statistics $\mathbf{T}(\rho) = \{\rho_i, \rho_i\rho_j, \rho_i\rho_j\rho_k\}$. This upstream population ρ expands noiselessly and linearly into the downstream population $\mathbf{r}$ according to $\mathbf{r} = A\rho$, where A is a 100×15 matrix whose elements are generated from a standard normal distribution. Because this expansion is linear, the sufficient statistics for $p(\mathbf{r}|s)$ are still polynomials up to third-order, $\mathbf{R}(\mathbf{r}) = \{r_i, r_ir_j, r_ir_jr_k\}$.

To compute the nonlinear information content of the downstream neurons, we estimate the stimulus from polynomial nonlinearities of $\mathbf{r}$. We first generate estimates $\hat{s}_1$ from $\mathbf{R}^{(1)} = \mathbf{r}$ directly. Then, to isolate information of second order, we removed this first-order information by subtracting the conditional mean responses $\langle r_i|\hat{s}_1\rangle$ given the first-order estimates from the full population (even unrecorded neurons) to obtain $\delta r_i = r_i - \langle r_i|\hat{s}_1\rangle$, and then compute second-order products $\mathbf{R}^{(2)} = \{\delta r_i \delta r_j\}$. To isolate third-order information, we remove the stimulus-dependent covariance $\Sigma_\mathbf{r}(\hat{s}_2) = \left\langle \delta\mathbf{r}\,\delta\mathbf{r}^\top|\hat{s}_2\right\rangle$ with estimates $\hat{s}_2$ based on a quadratic decoder from the full population, to obtain whitened deviations $\mathbf{z} = \Sigma_\mathbf{r}(\hat{s}_2)^{-1}\delta\mathbf{r}$, and evaluate their skewness as $\mathbf{R}^{(3)} = \{z_iz_jz_k\}$. This approach is natural because it is essentially the inverse of the generation process we used for quadratic and cubic codes (Section **Quadratic encoding** and **Cubic encoding**). Note that this isolation is not necessary to evaluate the nonlinear information content or to apply our choice correlation test, but here it allows us to identify how information in different nonlinear orders scales with population size.

We then construct estimates from random subsets of these statistics and approximate their Fisher information content by their inverse variance over trials with the same stimulus, $\mathbf{R}^{(z)}_{\text{subset}}$, $J_{\mathbf{R}^{(z)}_{\text{subset}}} = 1/\sigma^2_{\hat{s}_z^{\text{subset}}}$. Figure S6A shows the fraction of the information extracted from the selected subset of statistics to the information in all of the z-th order statistics, $J_{\mathbf{R}^{(z)}_{\text{subset}}}/J_{\mathbf{T}_z}$, plotted against the number of the decoded units. From the simulation, we find that the information ratio generically saturates at 1 after decoding M^z units, so we do not need to decode all N^z statistics of order z from the large downstream population. If the cortical expansion introduces noise, then the information will not

have completely saturated by M^z statistics, but most of the information will be extracted by recordings of that size [3].

In principle, the number of recorded units might also affect the accuracy of our nonlinear choice correlation test in assessing decoding efficiency. Figure S6B plots measured versus predicted linear, quadratic, and cubic choice correlations. Color shading indicates different numbers of decoded statistics, as indicated in Figure S6A. We found that in this testing regime the decoding efficiency revealed by the slope in the choice correlation test is not substantially affected by changing the number of measured statistics.

S.7.2 Assessing optimal decoding from estimation versus discrimination

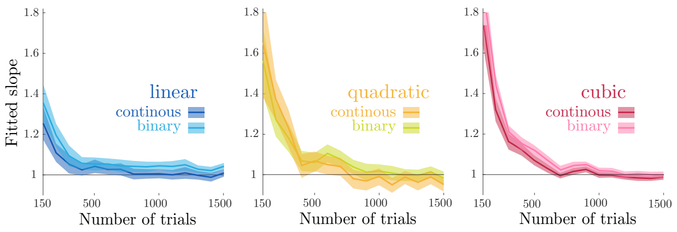

Figure S7: Optimal decoding can be revealed with equal fidelity from either fine continuous estimation or fine binary discrimination. We simulate neurons from random quadratic codes and decode them optimally to generate continuous estimates or binary choices. The three panels plot the slope of the relationship between predicted and measured nonlinear choice correlations, as a function of the number of trials. Different panels and colors denote the nonlinear types and task types. Means (denoted by solid lines) and 95% confidence interval (denoted by shades) for fitted slopes were compute by repeating the procedures for 30 times independently.

Here we use simulations to compare the outcomes of the nonlinear choice correlation test under continuous estimation versus binary discrimination. This simulation is based on quadratic codes (Supplemental Materials S.1.3) with 10 neurons whose response means and covariances contain information about the stimulus.

For the continuous estimation task, we assume the brain decodes neural activity through a weighted sum of linear and quadratic statistics $\mathbf{R}(\mathbf{r})$ minimizing the variance of an locally unbiased decoder, $\hat{s} = \mathbf{w}^\top \mathbf{R}(\mathbf{r}) + c$, with weights $\mathbf{w} \propto \Gamma^{-1}\mathbf{F}'$. Nonlinear choice correlations are measured directly in simulation, and the optimal decoding predictions are calculated by $C^{\text{opt}}_{R_k,\hat{s}} = \sqrt{\sigma^2_{\hat{s}}/\sigma^2_{\hat{s},R_k}}$ (Eq. 19), where $\sigma^2_{\hat{s}}$ is the variance of the locally optimal unbiased estimator based on the entire population, and $\sigma^2_{\hat{s},R_k}$ is the same for an estimator built only from R_k.

For binary discrimination, we assume the brain decodes neural activity using the same optimal decoding weights as used for the continuous estimation, but we now threshold it at a reference s_0 to obtain a binary output choice, $\hat{s}_\pm = \text{sgn}(\hat{s} - s_0)$. The measured choice correlations are now the correlations between the binary choice and nonlinear neural statistics. The optimal choice correlations are calculated by $C^{\text{opt}}_{R_k,\hat{s}_\pm} = \zeta^{\text{fine}} d'_{R_k}/d'$, where d' are sensitivities of the discriminators derived from the full population or just from R_k, and ζ^{fine} is the correction factor defined in Eqs. 152 and 154.

We then test the accuracy of the nonlinear choice correlation test in the two task settings while varying the number of trials. Figure S7 shows that the fitted slope between the measured and predicted choice correlations is biased to give a larger slope when there are few trials, with a slightly greater bias in binary discrimination than continuous estimation. These results reveal that binary tasks provide similar utility as continuous estimation in these settings.

References

[1] Shamir M, Sompolinsky H (2004) Nonlinear population codes. Neural computation 16: 1105–1136.

[2] Ecker AS, Berens P, Tolias AS, Bethge M (2011) The effect of noise correlations in populations of diversely tuned neurons. Journal of Neuroscience 31: 14272–14283.

[3] Moreno-Bote R, Beck J, Kanitscheider I, Pitkow X, Latham P, Pouget A (2014) Information-limiting correlations. Nature neuroscience 17: 1410–1417.

[4] Ecker AS, Berens P, Keliris GA, Bethge M, Logothetis NK, Tolias AS (2010) Decorrelated neuronal firing in cortical microcircuits. science 327: 584–587.

[5] Beck J, Bejjanki VR, Pouget A (2011) Insights from a simple expression for linear fisher information in a recurrently connected population of spiking neurons. Neural computation 23: 1484–1502.

[6] Paradiso M (1988) A theory for the use of visual orientation information which exploits the colum-

nar structure of striate cortex. Biological cybernetics 58: 35–49.

[7] Zohary E, Shadlen MN, Newsome WT (1994) Correlated neuronal discharge rate and its implications for psychophysical performance. Nature 370: 140–143.

[8] Sompolinsky H, Yoon H, Kang K, Shamir M (2001) Population coding in neuronal systems with correlated noise. Physical Review E 64: 051904.

[9] Pitkow X, Liu S, Angelaki DE, DeAngelis GC, Pouget A (2015) How can single sensory neurons predict behavior? Neuron 87: 411–423.

[10] Britten KH, Newsome WT, Shadlen MN, Celebrini S, Movshon JA (1996) A relationship between behavioral choice and the visual responses of neurons in macaque mt. Visual neuroscience 13: 87–100.

[11] Kanitscheider I, Coen-Cagli R, Pouget A (2015) Origin of information-limiting noise correlations. Proceedings of the National Academy of Sciences 112: E6973–E6982.

[12] Bethge M, Rotermund D, Pawelzik K (2002) Optimal short-term population coding: when fisher information fails. Neural computation 14: 2317–2351.

[13] Kang I, Maunsell JH (2012) Potential confounds in estimating trial-to-trial correlations between neuronal response and behavior using choice probabilities. Journal of neurophysiology 108: 3403–3415.
