# Supplementary material for: Revealing nonlinear neural decoding by analyzing choices
Source: Nat Commun. 2021 Nov 16;12:6557. doi: 10.1038/s41467-021-26793-9 (PMC8595442; doi:10.1038/s41467-021-26793-9)
Supplement: Supplementary file 3 — Reporting Summary [file 41467_2021_26793_MOESM3_ESM.pdf]

nature research

Corresponding author(s): Xaq Pitkow

Last updated by author(s): September 2021

Reporting Summary

Nature Research wishes to improve the reproducibility of the work that we publish. This form provides structure for consistency and transparency in reporting. For further information on Nature Research policies, see Authors & Referees and the Editorial Policy Checklist .

Statistics

For all statistical analyses, confirm that the following items are present in the figure legend, table legend, main text, or Methods section.

n/a	Confirmed	
☐	☒	The exact sample size (*n*) for each experimental group/condition, given as a discrete number and unit of measurement
☐	☒	A statement on whether measurements were taken from distinct samples or whether the same sample was measured repeatedly
☐	☒	The statistical test(s) used AND whether they are one- or two-sided<br>*Only common tests should be described solely by name; describe more complex techniques in the Methods section.*
☐	☒	A description of all covariates tested
☐	☒	A description of any assumptions or corrections, such as tests of normality and adjustment for multiple comparisons
☐	☒	A full description of the statistical parameters including central tendency (e.g. means) or other basic estimates (e.g. regression coefficient) AND variation (e.g. standard deviation) or associated estimates of uncertainty (e.g. confidence intervals)
☐	☒	For null hypothesis testing, the test statistic (e.g. *F*, *t*, *r*) with confidence intervals, effect sizes, degrees of freedom and *P* value noted<br>*Give P values as exact values whenever suitable.*
☒	☐	For Bayesian analysis, information on the choice of priors and Markov chain Monte Carlo settings
☒	☐	For hierarchical and complex designs, identification of the appropriate level for tests and full reporting of outcomes
☐	☒	Estimates of effect sizes (e.g. Cohen's *d*, Pearson's *r*), indicating how they were calculated

Our web collection on statistics for biologists contains articles on many of the points above.

Software and code

Policy information about availability of computer code

Data collection	All custom LabVIEW and MATLAB code used for electrophysiology data collection and data processing are made publicly available at the Tolias lab GitHub organization page https://github.com/atlab.
Data analysis	Code used for analysis and figure generation will be viewable and available for download from https://github.com/xaqlab/nonlinear_choice_correlation.

For manuscripts utilizing custom algorithms or software that are central to the research but not yet described in published literature, software must be made available to editors/reviewers. We strongly encourage code deposition in a community repository (e.g. GitHub). See the Nature Research guidelines for submitting code & software for further information.

Data

Policy information about availability of data

All manuscripts must include a data availability statement. This statement should provide the following information, where applicable:

- Accession codes, unique identifiers, or web links for publicly available datasets
- A list of figures that have associated raw data
- A description of any restrictions on data availability

Figure 6 was generated from processed electrophysiological data. The datasets generated and/or analyzed during the current study are available from the corresponding author upon reasonable request. No publicly available datasets were used in this study.

Field-specific reporting

Please select the one below that is the best fit for your research. If you are not sure, read the appropriate sections before making your selection.

Life sciences study design

All studies must disclose on these points even when the disclosure is negative.

Sample size	The sample size of two monkeys has been established following the standard practice in the non-human primate research and to ensure independent replication of results observed from the first monkey in the second monkey.
Data exclusions	Data were excluded from sessions when the monkey was not performing effectively, as described in the paper.
Replication	Every finding described in this study on one monkey has been successfully replicated on the second monkey that was independently trained, recorded and analyzed.
Randomization	There were no distinct experimental groups used in this study.
Blinding	There were no distinct experimental groups used in this study.

Reporting for specific materials, systems and methods

We require information from authors about some types of materials, experimental systems and methods used in many studies. Here, indicate whether each material, system or method listed is relevant to your study. If you are not sure if a list item applies to your research, read the appropriate section before selecting a response.

Materials & experimental systems

n/a	Involved in the study	
☒	☐	Antibodies
☒	☐	Eukaryotic cell lines
☒	☐	Palaeontology
☐	☒	Animals and other organisms
☒	☐	Human research participants
☒	☐	Clinical data

Methods

n/a	Involved in the study	
☒	☐	ChIP-seq
☒	☐	Flow cytometry
☒	☐	MRI-based neuroimaging

Animals and other organisms

Policy information about studies involving animals; ARRIVE guidelines recommended for reporting animal research

Laboratory animals	This study involved two healthy, male rhesus macaque (Macaca mulatta) monkeys aged 10 and 7 years.
Wild animals	The study did not involve wild animals.
Field-collected samples	No samples were collected from the field.
Ethics oversight	All procedures were approved by the Institutional Animal Care and Use Committee (IACUC) of Baylor College of Medicine.

Note that full information on the approval of the study protocol must also be provided in the manuscript.
